# A Review on Signal Processing Approaches to Reduce Calibration Time in EEG-Based Brain–Computer Interface

**DOI:** 10.3389/fnins.2021.733546

**Published:** 2021-08-19

**Authors:** Xin Huang, Yilu Xu, Jing Hua, Wenlong Yi, Hua Yin, Ronghua Hu, Shiyi Wang

**Affiliations:** ^1^Software College, Jiangxi Normal University, Nanchang, China; ^2^School of Software, Jiangxi Agricultural University, Nanchang, China; ^3^School of Mechatronics Engineering, Nanchang University, Nanchang, China; ^4^Youth League Committee, Jiangxi University of Traditional Chinese Medicine, Nanchang, China

**Keywords:** signal processing, transfer learning, semi-supervised learning, EEG, brain–computer interface, calibration

## Abstract

In an electroencephalogram- (EEG-) based brain–computer interface (BCI), a subject can directly communicate with an electronic device using his EEG signals in a safe and convenient way. However, the sensitivity to noise/artifact and the non-stationarity of EEG signals result in high inter-subject/session variability. Therefore, each subject usually spends long and tedious calibration time in building a subject-specific classifier. To solve this problem, we review existing signal processing approaches, including transfer learning (TL), semi-supervised learning (SSL), and a combination of TL and SSL. Cross-subject TL can transfer amounts of labeled samples from different source subjects for the target subject. Moreover, Cross-session/task/device TL can reduce the calibration time of the subject for the target session, task, or device by importing the labeled samples from the source sessions, tasks, or devices. SSL simultaneously utilizes the labeled and unlabeled samples from the target subject. The combination of TL and SSL can take advantage of each other. For each kind of signal processing approaches, we introduce their concepts and representative methods. The experimental results show that TL, SSL, and their combination can obtain good classification performance by effectively utilizing the samples available. In the end, we draw a conclusion and point to research directions in the future.

## Introduction

A brain–computer interface (BCI) can allow a subject to directly control an external electronic device using his brain signals without the participation of his peripheral nerves and muscles (Vidal, 1977; Kübler et al., 2001; Wolpaw et al., 2002). BCI technology can not only help the patients suffering from neuromuscular diseases, such as amyotrophic lateral sclerosis (ALS), recover or live independently, but also provide the healthy subjects with a novel way to communicate with the external environment (Chai et al., 2014; Horki et al., 2015). Therefore, BCI technology can play an important role in rehabilitation engineering, military, and entertainment (Ma et al., 2015; Soekadar et al., 2016).

So far, there are three categories of BCIs divided by the extent of invading the brain, including non-invasive BCIs, semi-invasive BCIs, and invasive BCIs (Rao, 2010). Due to safety, non-invasive BCIs have drawn increasing attention, which can collect non-invasive brain signals, such as electroencephalograms (EEGs), functional magnetic resonance imaging (fMRI), and functional near-infrared spectroscopy (fNIRS). Particularly, due to low cost and high temporal resolution, EEG signals are widely used in the non-invasive BCIs and much suitable for real-time BCI control.

However, EEG signals are weak, sensitive to noise/artifact, and non-stationary. It is challenging to classify EEG signals accurately. Moreover, it is difficult for a subject to freely control his brain activity to execute kinds of BCI tasks. On past decades, three classic EEG paradigms have been widely studied as follows:

(1) Motor imagery (MI) (Pfurtscheller and Neuper, 2001), which is a typical BCI paradigm, was firstly designed for rehabilitation treatment. The individuals with neuromuscular diseases are expected to recover their damaged motor nerves by effectively implementing MI tasks. Unfortunately, people are familiar with real movement naturally instructed by their brain activity, instead of movement imagination. Therefore, MI EEG signals are quite unsteady, limiting the categories of MI. The mostly used MI tasks include left hand, right hand, foot, and tongue MI (Kumar and Inbarani, 2017; She et al., 2020).
(2) Event-related potentials (ERP) (Blankertz et al., 2011; Zhang et al., 2012), which are invoked by an attended stimulus, have more obvious features than MI EEG signals. For example, the P300 ERP can be detected after a rare and relevant stimulus onset, with a latency around 300 ms. Farwell and Donchin (1988) demonstrated a P300 speller system with 36 commands and an information transfer rate (ITR) of less than 30 bits/min.
(3) Steady-state visual evoked potentials (SSVEP), which are evoked by a low-frequency flickering stimulus, oscillate at a multiple of the stimulus frequency (Yin et al., 2015; Adair et al., 2017; Chiang et al., 2019). SSVEP-based BCI has advantages, such as multiple commands, high classification performance, and high ITR. However, users may suffer from visual fatigue after long-term gazing at the flickering stimulus.

**FIGURE 1:**
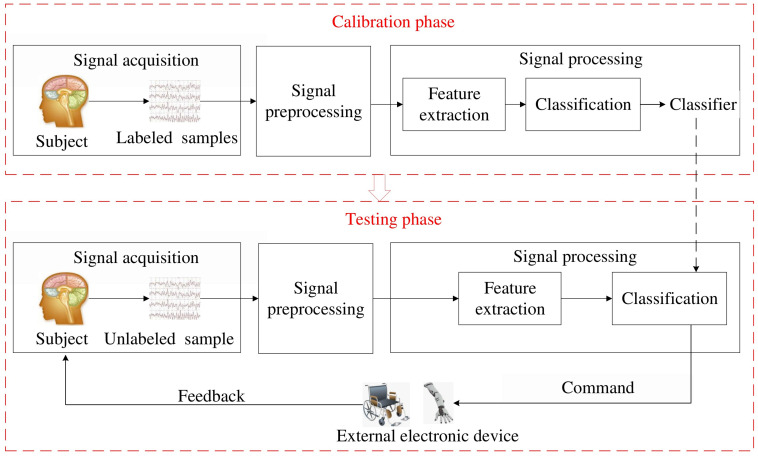
The whole process of EEG-based BCI system.

Besides these traditional paradigms, some meaningful paradigms become emerging in EEG-based BCI. For instance, emotion states (happy, neutral, and sad) can be detected in EEG-based affective BCI (Muhl et al., 2014; Shen and Lin, 2019). Drive drowsiness estimation from EEG signals can be used to avoid traffic accidents (Wei et al., 2015; Wu et al., 2016; Chai et al., 2017). Although EEG-based BCI has promising perspective, it must deal with the weakness of EEG and uncontrollability of BCI task. In this paper, we discuss the signal processing approaches to tackle this problem. A whole process of a traditional EEG-based BCI system is illustrated in Figure 1.

In Figure 1, the whole process is comprised of two consecutive phases: calibration phase and testing phase. In the calibration phase, labeled samples from each subject are successively processed by different modules to train a subject-specific classifier. Then, in the testing phase, an unlabeled sample is identified by the tailored classifier and translated into a command to control an external electronic device, e.g., a wheelchair or an exoskeleton arm. Here, we briefly introduce main modules in both phases as follows:

(1) Signal acquisition, which is used to acquire the EEG signals using multiple dry/wet electrodes and then digitize these electrical signals. It is a critical step for BCI systems. In the calibration phase, the raw EEG signals are invoked by assigned BCI tasks. A reliable classifier benefits from amounts of labeled samples. In the testing phase, the raw EEG signals are invoked by unknown human intentions and then identified in the classification module.
(2) Signal preprocessing, which aims to improve signal quality without losing important information. In this step, the raw EEG signals, easily contaminated by electrooculograms (EOGs), electromyograms (EMGs), and DC drift, are cleaned and denoized to enhance their relevant information. Principal component analysis (PCA) and independent component analysis (ICA) are often used to remove EMGs and EOGs from the raw EEG signals.
(3) Feature extraction, which aims at extracting a few relevant features from the preprocessed EEG signals to alleviate the computational burden of classification. The brain patterns can be characterized by these features. The preprocessed EEG signals are typically filtered in the time domain (band-pass filter), frequency domain, time-frequency domain, or spatial domain (spatial filter). The best filters optimized in the calibration phase are used to extract valuable features in the testing phase.
(4) Classification. In the calibration phase, a set of labeled features are input to the classification module to build a subject-specific classifier. In the testing phase, an unlabeled feature is assigned to a class by this tailored classifier. A class corresponds to one type of BCI tasks.

The neurophysiological processes often vary over time and across subjects in most EEG-based BCIs. For each subject, long calibration procedure is always needed to collect amounts of labeled EEG signals to build a steady recognition model. However, tedious calibration may cause user’s mental exhaustion and degenerate the experimental effect. It is hard to get abundant EEG signals for each subject. In Table 1, some publicly available EEG-based datasets are listed.

**TABLE 1:** Publicly available EEG-based datasets.

Dataset	Subject	Task	Total trials (per subject)	References
Dataset A/B	10/9	Left/right hand MI	300/90	Alamgir et al. (2010)
BCI III IVa	5	Right hand/foot MI	280	Blankertz et al. (2006)
BCI IV IIa	9	Left/right hand, foot, and tongue MI	288	Keng et al. (2012)
MI BCI	52	Left/right hand MI	200 or 240	Hohyun et al. (2017)
P300 speller	8	36 characters	35	Riccio et al. (2013)
SEED	15	Negative, neutral, and positive emotion states	15	Zheng and Lu (2015)

As shown in Table 1, the number of total EEG trials is limited for each subject in most datasets. Accordingly, labeled samples are not enough to be exploited. Therefore, it is crucial to make full use of the samples available and face up to the differences among them. For each subject, the lack of labeled samples motivates the approaches that go beyond traditional supervised learning by importing labeled samples from other sessions/subjects/tasks/devices, unlabeled samples from himself, and even artificial samples (Lotte, 2015). These approaches include transfer learning (TL), deep learning (DL), semi-supervised learning (SSL), artificial data generation and so on.

Transfer learning is a popular machine learning technique to shorten calibration time because it transfers abundant labeled samples from different sessions/subjects/tasks/devices, denoted as source domains, to a new session/subject/task/device, denoted as a target domain. The main hypothesis in TL is that the target and source domains belong to the same feature space. However, high inter-domain variability often makes the hypothesis violated. It is key to effectively integrate the features of the source domains with those of the target domain.

Deep learning is a promising subfield of machine learning, which has successful applications in many fields, such as natural language processing and computer vision, etc (Huang et al., 2021). Recently, it has attracted more attention in BCIs (Dose et al., 2018; Ming et al., 2018; Liu Y. et al., 2020; Zhang Y. et al., 2020). It can build a unified end-to-end model directly applied to raw EEG signals. However, its good performance strongly relies on sufficient labeled samples and a huge computational cost. Although DL cannot directly shorten the calibration time for a new BCI subject, its pre-trained model trained from a large scale of source domains can improve the performance of the BCI system. In the Section of TL, we discuss a combination of DL and TL.

Semi-supervised learning can simultaneously use the labeled and unlabeled samples from the same subject to train a classifier. For offline SSL, the inherent information embedded in the relatively large unlabeled set can effectively make up the insufficiency of the labeled set. For online SSL, the classifier can be updated in real time using a small, labeled set and an increasing unlabeled set with pseudo labels. To address with-in subject variability, SSL works by making the following assumptions, e.g., the smoothness assumption, the cluster assumption, and the low-density assumption. In general, with-in subject variability in SSL should be less than the inter-domain variability in TL. Therefore, it is valuable for SSL to reduce calibration time.

Recently, a combination of TL and SSL has been popular since it can take advantage of TL and SSL simultaneously. On the one hand, it can make up the shortage of the labeled samples by using TL. On the other hand, it can train a more subject-specific model by importing the unlabeled samples from the target subject.

Artificial data generation is also a good choice to cope with the shortage of the labeled samples. Lotte (2015) generated numerous artificial labeled samples from the few original labeled samples available for EEG-based BCI by means of segmentation and recombination in the time domain or time-frequency domain. In the dataset 1 of BCI competition IV, a challenging problem was posed to discriminate between the artificial data and real data (Keng et al., 2012). The performance of artificial data generation strongly relies on the number and quality of original labeled data.

In this paper, we focus on commonly used approaches to shorten calibration effort, such as TL and SSL. Generally, TL and SSL are often used in feature extraction and classification, respectively. To our best knowledge, there is no comprehensive review on signal processing approaches to reduce calibration time in EEG-based BCI. Lotte (2015) performed a short review on the existing signal processing approaches to shorten or suppress calibration time and paid more attention to the introduction to their new methods. There are comprehensive reviews on the application of TL in EEG-based BCI (Wang et al., 2015; Zhang K. et al., 2020). However, few reviews on SSL are referred.

In this paper, we give an overview of signal approaches to minimize calibration time in EEG-based BCI. Meanwhile, the experimental results of representative approaches are analyzed. BCI illiteracy means that the classification accuracy of subject cannot reach 70%. To avoid BCI illiteracy, approximately 40 labeled samples per class are required for BCI subject, as suggested in Blankertz et al. (2007). Therefore, it is crucial for signal processing approaches to make full use of the data available to help the subject obtain satisfied performance using his labeled samples as few as possible.

The remainder of this paper is organized as follows. In Sections “Transfer Learning” and “Semi-supervised Learning,” TL and SSL approaches are reviewed, respectively. In Section “A Combination of Transfer Learning and Semi-Supervised Learning,” a combination of TL and SSL is introduced. A discussion of different signal processing approaches is presented in Section “Discussion.” Finally, a conclusion is drawn in Section “Conclusion.”

## Transfer Learning

### Concepts

Transfer learning is usually designed to cope with the shortage of the labeled data from the target domain by transferring the labeled data from other source domains. As mentioned above, a domain refers to a session, a subject, a task, or a device, etc. However, high between-domain variability in EEG-based BCI raises more problems: how to evaluate, minimize, and utilize such differences? To address these problems, we introduce the formal definition of TL and discuss state-of-the-art TL methods.

**Definition 1**. As in Pan and Yang (2010), a domain 𝒟 consists of a feature space 𝒳 and its marginal probability distribution *P*(*X*), where *X* ∈ 𝒳. A task 𝒯 is defined as a label space 𝒴 and a predictive function *f*(*X*). Although a source domain 𝒟_*s*_ do not match a target domain 𝒟_*T*_, or a source task 𝒯_*s*_ is different from a target task 𝒯_*T*_, i.e., 𝒟_*s*_≠𝒟_*T*_, or 𝒯_*s*_≠𝒯_*T*_, TL still aims to improve the learning level of the target predictive function *f*_*T*_(*X*) by adding the knowledge embedded in *N_s_* source domains ${\left\{{𝒟}_{s}^{i}\right\}}_{i=1}^{N_{s}}$ and their associated tasks ${\left\{{𝒯}_{s}^{i}\right\}}_{i=1}^{N_{s}}$.

In this paper, state-of-the-art TL approaches in EEG-based BCI are categorized into three groups based on “What knowledge should be transferred” as follows (Pan and Yang, 2010):

(1) Instance transfer learning (ITL), which directly transfers the certain parts of the data (instance) in the source domains by reweighting. It is crucial to evaluate the similarity between the source and target domains and convert the corresponding similarity metric into optimal weights used in the source and target domains.
(2) Feature-representation transfer (FRT), which transfers the stationary feature representation from the source domains and adjusts the discriminative feature representation for the target domain. Unlike ITL, FRT learns “good” feature representations from the data in the source domains.
(3) Parameter transfer learning (PTL), which transfers the parameters of the model learning from the source domains based on the assumption that the source and target domains share these parameters. Then, PTL updates the parameters for the target domain.

In the following, we introduce some typical TL approaches belonging to different groups for analysis.

### Instance Transfer Learning

In EEG-based BCI, ITL is widely used since it is easy to implement. In the phase of feature extraction, the samples from the source and target domains are usually reweighted based on the filters. Additionally, data alignment schemes have promising perspectives to minimize the differences between domains. Therefore, we introduce the filter-based ITL and data alignment-based ITL, respectively.

#### Filter-Based Instance Transfer Learning

Kullback-Leibler (KL) divergence is always used to compute similarities between two sets of EEG data. In weighted logistic regression-based TL (wLRTL) algorithm, the common spatial patterns (CSP) filtered EEG samples from each source subject were weighted based on KL divergence between the source and target subjects in a supervised way or an unsupervised way (Azab et al., 2019). In the supervised case, the divergence of each source subject is equal to the average of the KL divergences computed for each class. In the unsupervised case, the divergence of each source subject is calculated without using the class labels. Then, the regularization parameter calculated using the divergences of all source subjects is added to the objective function of logistic regression classifier to make full use of the labeled samples from the source subjects correctly. Cao et al. (2021) verified the applicability of wLRTL approach for stroke patients and intra/inter- subjective conditions.

Bhattacharyya distance was used to measure similarities between the source and target datasets based on their class conditional distributions in a probabilistic TL approach (Khalaf and Akcakaya, 2020). First, the CSP features of MI EEG trials from the source datasets are converted into scalar support vector machine (SVM) scores to obtain their class conditional distributions. Then, the similarity metrics of different source datasets are identified based on these class conditional distributions using Bhattacharyya distance. Finally, the top similar source datasets are combined with the target dataset to train the classifier.

Regularized CSP (RCSP) approaches have been very promising for cross-subject TL in the small-sample setting (Wang and Li, 2016). Based on the framework of RCSP, weighted covariance matrices of EEG data from the source and target subjects are simultaneously utilized to obtain optimal CSP filters. The distance metrics, including KL divergence, Frobenius norm, cosine distance, are used to measure the similarity between the source and target data (Kang et al., 2009; Cheng et al., 2017; Xu et al., 2019b).

A RCSP based on dynamic time warping (DTW-RCSP) approach was proposed to shorten temporal variations and non-stationarities between the subjects by aligning the labeled samples from all source subjects to the average of few target samples from the same class using an optimal warping path (Azab et al., 2020). This warping path is computed using dynamic programming. Then, using different weights, the subject-specific covariance matrix estimated using the labeled samples from the target subject is combined with the DTW-based transferred covariance matrix estimated using the aligned labeled samples from all source subjects mentioned above to get best CSP filters for the target subject.

The objective functions are often optimized to gain a weighted/projecting matrix/vector which can minimize the differences among domains (Dai et al., 2018; Zou et al., 2018; Jiao et al., 2019).

Zou et al. (2018) proposed two data mapping methods to diminish the inter-subject variation as much as possible.

In the first data mapping method, the transformation matrix ℒ is optimized to reduce the inter-subject variation caused by dissimilarities in channel locations as follows:

(1) $$\underset{ℒ}{\text{argmin}}\left({∥W_{1}-W_{2}\,{ℒ}^{T}∥}_{F}^{2}+{∥ℒ-P∥}_{F}^{2}\right),s\,t.{ℒ}^{T}\,ℒ=I$$

where *W_1_* and *W_2_* are the CSP filters calculated from the target domain *D_1_* and the source domain *D_2_*, respectively. A new form $D_{2}^{\prime }$ which has similar channel locations with *D_1_* is generated by projecting ℒ to original source domain *D_2_*, i.e., $D_{2}^{\prime }=ℒ\,D_{2}$. *P* is the constraint matrix with the element *P*_*ij*_ = *e*^−*d*(*i*,*j*)^, where *d*(*i*,*j*) is the distance between the channel *i* and the channel *j*. *I* is the identity matrix.

In the second data mapping method, the inter-subject variation of intensity of event-related desynchronization/synchronization (ERD/ERS) is minimized. CSP is designed to find the variance of the EEG signals between different subjects, instead of different classes. Therefore, the eigenvector of the CSP filter matrix is used to generate a correction matrix which can adjust the source data based on the target data.

Jiao et al. (2019) proposed a sparse group representation model (SGRM), which tried to seek the optimal representation vector for an unlabeled testing sample from the target subject by effectively using the labeled training samples from the target and source subjects. Each subject is regarded as a group. SGRM can realize not only the group-wise sparsity with the *L*_2,1_-norm but also within-group sparsity with the *L*_1_-norm. The representation vector *u*^∗^ is optimized as:

(2) $$\underset{u^{*}}{\text{argmin}}\left(\frac{1}{2}\,{∥\tilde{G}\,u^{*}-\hat{g}∥}_{2}^{2}+\lambda_{1}\,{∥u^{*}∥}_{1}+\lambda_{2}\,\left({∥u_{0}∥}_{2}+\sum_{i=1}^{N_{s}}{∥u_{i}∥}_{2}\right)\right),$$

where $\hat{g}$ denotes the CSP feature of the testing sample, $\tilde{G}$ concatenates the dictionary matrices from all subjects. The regularization terms λ_1_ and λ_2_ aim to control the within-group sparsity and the between-group sparsity, respectively. *N_s_* denotes the number of source subjects. *u_0_* and *u_i_* correspond to the sparse representation vectors of the target subject and the *i*-th source subject, respectively.

#### Data Alignment-Based Instance Transfer Learning

Recently, Riemannian alignment-based ITL approaches have drawn increasing attention in EEG-based BCI (Barachant et al., 2013; Qi et al., 2018; Zanini et al., 2018; Singh et al., 2019; Zhang and Wu, 2020). The reason is that the affine transformation makes data from different domains similar.

Covariance matrix is symmetric and positive semi-definite. It is positive definite when the sampling rate is high enough (Barachant et al., 2013). It is shown that symmetric positive definite (SPD) matrices belong to the smooth Riemannian manifold (Moakher, 2006). Therefore, as EEG features, covariance matrices are often used in the Riemannian manifold.

**FIGURE 2:**
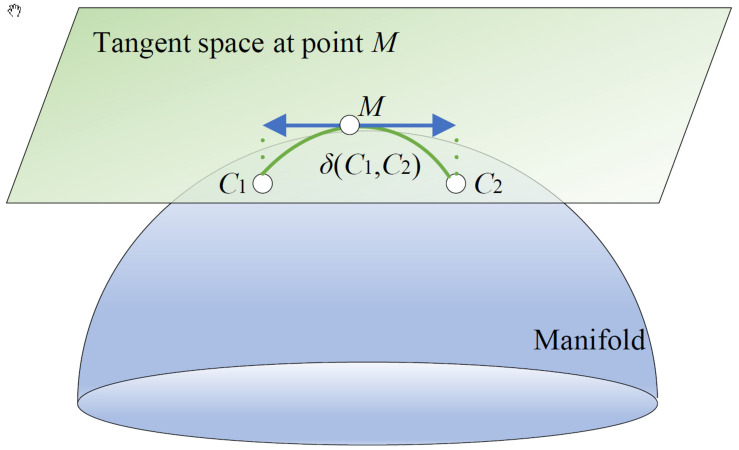
A Riemannian manifold and its tangent space.

In Figure 2, the covariance matrices *C_1_* and *C_2_* are the two points of the Riemannian manifold. According to the congruence invariance property, the Riemannian distance δ(*C*_1_,*C*_2_)between *C_1_* and *C_2_* is invariant with respect to a change of reference matrix *M*. In general, *M* is the Riemannian mean of a specific domain, e.g., a class, a session, or a subject, etc.

After choosing *M* as the reference matrix, the original covariance matrices ${\left\{C_{i}^{\kappa }\right\}}_{i=1}^{N_{\kappa }}$ from the κ-th domain with *N*_κ_ trials are converted into aligned ones, i.e., ${\left\{{\tilde{C}}_{i}^{\kappa }=M^{-1/2}C_{i}^{\kappa }M^{-1/2}\right\}}_{i=1}^{N_{\kappa }}$ which center on the identity matrix. Each aligned covariance matrix is approximately an identity matrix, which can shorten the differences of each other.

A Riemannian geometry cross-session/subject TL framework was built in MI-based and P300-based BCIs (Zanini et al., 2018).

Firstly, some resting-state signals in each domain are collected to calculate their Riemannian mean. The resulting Riemannian mean is assigned as the reference matrix of corresponding domain. In MI-based BCI, resting signals are extracted from time windows of 1.5 s, where no BCI tasks are performed. However, in P300-based BCI, since there is not a separated resting-state signal, reference matrix is calculated using the non-target trials.

Then, after Riemannian alignment (RA), the original covariance matrices from each domain are converted into aligned ones using the reference matrix of corresponding domain. All aligned labeled covariance matrices from source domains are used to estimate the centers of different classes and train a minimum distance to mean (MDM) classifier based on Riemannian Gaussian distributions.

Finally, the aligned testing covariance matrix from the target domain can be assigned to a class by the proposed classifier, considering its Riemannian distance to the center of class and the maximum likelihood estimator of the dispersion parameter of the class simultaneously.

Zhang and Wu (2020) proposed a cross-subject manifold embedded knowledge transfer (MEKT) approach. It is challenging and meaningful for MEKT to transfer the labeled source domains into the unlabeled target domain, which can boost zero-training for a new subject. Three steps of MEKT are briefly introduced as follows:

(1) Covariance matrix centroid alignment: MEKT separately performs RA for the covariance matrices in the source and target domains using their Riemannian means as corresponding reference matrices, so that the marginal probability distributions from different domains are similar.
(2) Tangent space (TS) feature extraction: MEKT converts the aligned covariance matrices from different domains into the tangent space feature vectors. Then, MEKT assembles the tangent feature vectors from the source domains.
(3) Mapping matrices identification: MEKT finds optimal projection matrices *A* for the labeled source feature vectors *X_s_* and *B* for the unlabeled target feature vectors *X_T_* to minimize the joint probability distribution shift between domains.

Finally, a shrinkage linear discriminant analysis (shrinkage LDA, sLDA) classifier is trained using the labeled source feature vectors *A^T^X*_*s*_ and applied to the unlabeled target feature vectors *B^T^X*_*T*_ to predict their classes. To further avoid negative transfer, Zhang and Wu (2020) put forward a domain transferability estimation (DTE) approach based on MEKT, which selected most similar source subjects before performing the second step above. The transferability of the *i*-th source subject ${𝒟}_{s}^{i}$ is defined as follows:

(3) $$t\,r\,a\,n\,s\,f\,e\,r\,a\,b\,i\,l\,i\,t\,y\,\left({𝒟}_{s}^{i},{𝒟}_{T}\right)=\frac{{∥S_{b}^{{𝒟}_{s}^{i}}∥}_{1}}{{∥S_{b}^{{𝒟}_{s}^{i},{𝒟}_{T}}∥}_{1}},$$

where $S_{b}^{{𝒟}_{s}^{i}}$ denotes the between-class scatter matrix of ${𝒟}_{s}^{i}$, and $S_{b}^{{𝒟}_{s}^{i},{𝒟}_{T}}$ denotes the scatter matrix between the *i*-th source subject and the target subject 𝒟_*T*_. Then, the source subject with the highest transferability is chosen to be transferred.

Likewise, to select suitable source subjects, after RA, the aligned covariance matrices from a target subject are temporally used as the testing data and classified by an MDM classifier, which is trained using the aligned covariance matrices from a candidate source subject (Yong and Yu, 2020). The source subjects with higher classification performance are selected.

A Riemannian Procrustes analysis (RPA) approach was proposed using three geometrical transformations (translation, scaling, and rotation) in sequence (Rodrigues et al., 2019). During transformations, the covariance matrices from different domains have went through the steps of re-centering, stretching, and rotation. RPA is a typical semi-supervised TL method, utilizing the labeled data *C*_*sl*_ from the source domain, the labeled data *C*_*Tl*_ and the unlabeled data *C*_*Tu*_ from the target domain altogether.

In the translation phase, as in Zanini et al. (2018), the source labeled data *C*_*sl*_ and the target data *C*_*T*_ = *C*_*Tl*_∪*C*_*Tu*_ are separately re-centered around the identity matrix using the resting activity of each session to compute the Riemannian means of different domains.

Then, in the scaling phase, the re-centered source labeled data $C_{s\,l}^{\left(r\,c\,t\right)}$ and target labeled data $C_{T\,l}^{\left(r\,c\,t\right)}$are used to calculate the ratio of their dispersions, namely the scaling factor *r*. The stretched source labeled data $C_{s\,l}^{\left(s\,t\,r\right)}$ and target data are $C_{T}^{\left(s\,t\,r\right)}$ are formed using the above scaling factor *r*, i.e., $C_{s\,l}^{\left(s\,t\,r\right)}={\left(C_{s\,l}^{\left(r\,c\,t\right)}\right)}^{r}$, $C_{T}^{\left(s\,t\,r\right)}={\left(C_{T}^{\left(r\,c\,t\right)}\right)}^{r}$, where $C_{T}^{\left(r\,c\,t\right)}=C_{T\,l}^{\left(r\,c\,t\right)}\cup C_{T\,u}^{\left(r\,c\,t\right)}$. It should be noted that the re-centering and stretching transformations, without using the trials’ labels, work in an unsupervised way.

Finally, in the rotation phase, the trials’ labels are needed to compute an orthogonal rotation matrix *U*, which can be optimized to minimize the distance between the class mean of the source domain and that of the target domain. The rotated datasets are transformed as follows:

(4) $$C_{S\,l}^{\left(r\,o\,t\right)}=U^{T}\,C_{S\,l}^{\left(s\,t\,r\right)}\,U,C_{T}^{\left(r\,o\,t\right)}=U^{T}\,C_{T}^{\left(s\,t\,r\right)}\,U.$$

After three geometrical transformations for the covariance matrices from different domains, the MDM classifier is used to infer the label of the unlabeled data from the target domain.

Since most classifiers are designed for the Euclidean space, instead of the Riemannian space, Euclidean alignment (EA) extends RA in the Euclidean space using the Euclidean mean as the reference matrix (He and Wu, 2020b). For each subject, the original covariance matrices are transformed into the aligned ones using his resting activities to calculate his Euclidean mean. The concatenated aligned training covariance matrices from different source subjects are filtered by CSP and then trained by LDA. The CSP features of the target subject are extracted from their aligned covariance matrices by CSP and assigned to a class by LDA.

Mathematically, the Euclidean mean is the arithmetic mean of all covariance matrices. And the Riemannian mean can be computed by iterating the following three steps until convergence: converting all covariance matrices in the manifold into the tangent space feature vectors, calculating the arithmetic mean of such feature vectors, and converting the arithmetic mean back into the covariance matrix in the manifold. Apparently, EA is simpler and faster than RA.

**FIGURE 3:**
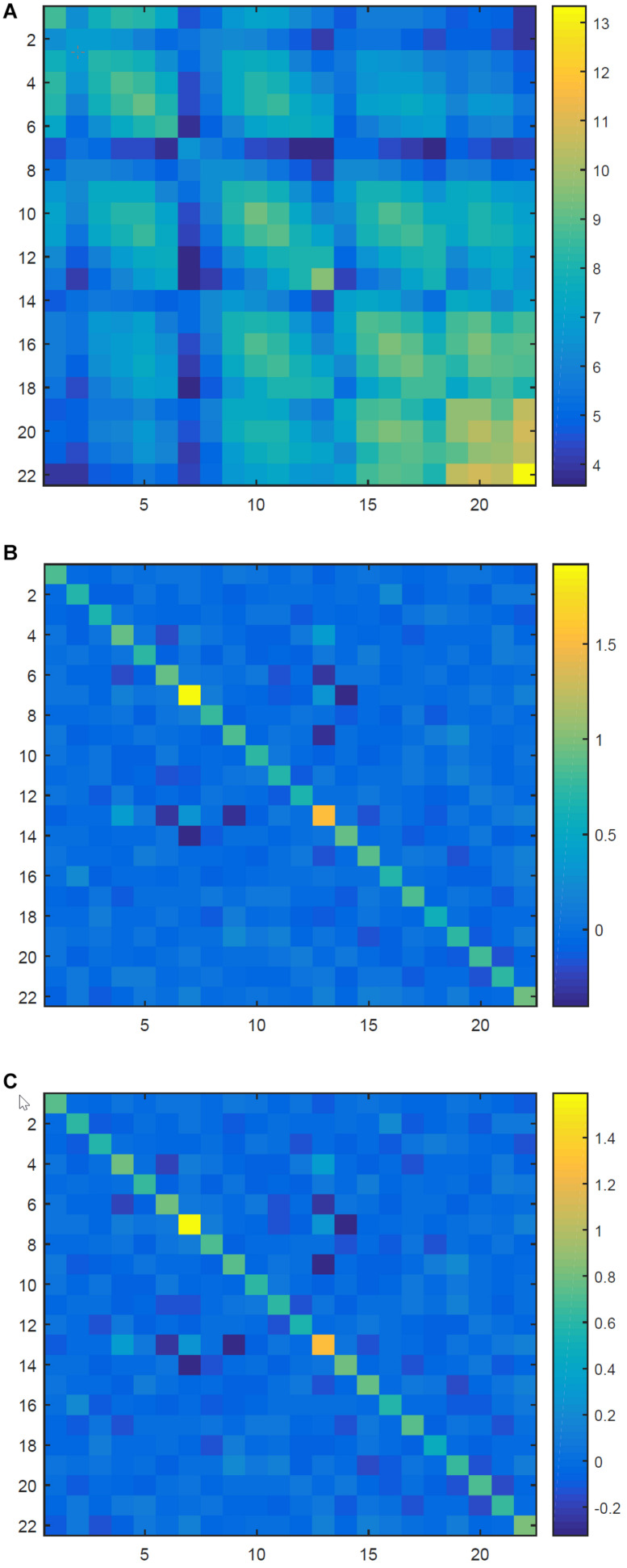
The raw covariance matrix and different aligned matrices. **(A)** The raw covariance matrix. **(B)** The aligned covariance matrix after RA. **(C)** The aligned covariance matrix after EA.

Furthermore, both RA and EA can make the aligned covariance matrices comparable since they are close to the identity matrix. To testify this, in Figure 3, we depict a raw covariance matrix and different aligned covariance matrices for the first EEG trial of A07 in dataset BCI IV IIa (Keng et al., 2012). As in Zhang and Wu (2020), this dataset contained 22-channel EEG recordings from nine healthy subjects. All raw EEG signals were band-pass filtered between 8 and 30 Hz using a causal 50-order finite impulse response filter. Then, the filtered signals were extracted from time segments ranging from 0.5 to 3.5 s. For each subject, their raw covariance matrices can be transformed into the aligned ones using Riemannian mean or Euclidean mean of their corresponding session as the reference matrix.

In Figure 3A, the raw covariance matrix is not like the identity matrix at all. However, as shown in Figures 3B,C, the aligned matrices are approximately identity matrix after RA and EA, respectively.

A label alignment (LA) was proposed to tackle cross-task transfer when the source and target subjects had different label spaces (He and Wu, 2020a). It is assumed that different subjects have the same number of classes. LA first performs EA for the per-class covariance matrices of each source subject. Then, LA builds *k*-medoids clustering on the unlabeled target domain based on a few labeled target trials and Riemannian distance. Then, LA labels the *k* cluster centers and performs EA for the per-class covariance matrices of the target subject. Next, LA matches each label in the source domain with one label in the target domain regularly or randomly, which depends on the similarity between label spaces. Finally, LA seeks a transformation matrix for each class of the source subject which can reduce the differences between the source subject’s covariance matrices belonging to the specific class and the target subject’s covariance matrices belonging to the matched class.

### Feature-Representation Transfer

The main idea of FRT is to transfer the stationary features across different source domains and adjust the discriminative features for the target domain (Sybeldon et al., 2017; Yin et al., 2017; Hossain et al., 2018; Jin et al., 2018).

So far, there are many TL approaches using spatial filters to generate feature representations for the raw EEG data (Nakanishi et al., 2019; Zheng X. et al., 2020; Hehenberger et al., 2021). Nakanishi et al. (2019) proposed a task-related component analysis (TRCA) approach in a cross-device SSVEP-based BCI speller system. TRCA consists of two steps: extracting task-related spatial filters for a source device and extracting task-unrelated spatial filters for a target device.

Step 1:

Task-related spatial filters are designed to maximize their reproducibility during the task periods. For each visual stimulus in SSVEP-based BCI, a specific task-related filter $\overset{`}{w}$ is optimized by maximizing the inter-trial correlation of its projections $z\,\left(t\right)={\overset{`}{w}}^{t}\,x\,\left(t\right)$, where *x*(*t*) ∈ *ℝ*^*N_c1_*^ denotes the *t*-th sample point of an observed EEG trial *x* collected from *N*_*c_1_*_ channels in a source device. Two devices have inconsistent electrode montages.

The covariance matrix *C*_*h_1_,h_2_*_ between the projection *z*^(*h*_1_)^ of the *h_1_*-th trial *x*^(*h*_1_)^ and the projection *z*^(*h*_2_)^ of the *h_2_*-th trial *x*^(*h*_2_)^ is defined as follows:

(5) $$C_{h_{1},h_{2}}=C\,o\,v\,\left(z^{\left(h_{1}\right)},z^{\left(h_{2}\right)}\right)=\sum_{k_{1},k_{2}=1}^{N_{c_{1}}}{\overset{`}{\omega }}_{k_{1}}\,{\overset{`}{\omega }}_{k_{2}}\,C\,o\,v\,\left(x_{k_{1}}^{\left(h_{1}\right)},x_{k_{2}}^{\left(h_{2}\right)}\right),$$

where ${\overset{`}{\omega }}_{k_{1}},{\overset{`}{\omega }}_{k_{2}}\in \overset{`}{w}$, *z*^(*h*_1_)^,*z*^(*h*_2_)^ ∈ *ℝ^N_sa_^* and *x*^(*h*_1_)^,*x*^(*h*_2_)^ ∈ *ℝ*^*N_c_1__*×*N*_*sa*_^, *N*_*sa*_ denotes the number of samples in each trial. Then, for the specific visual stimulus, all covariance matrices between different labeled training trials are summed as:

(6) $$\sum_{h_{1},h_{2}=1,h_{1}\neq h_{2}}^{N_{t\,r}}C_{h_{1},h_{2}}=\sum_{h_{1},h_{2}=1,h_{1}\neq h_{2}}^{N_{t\,r}}\sum_{k_{1},k_{2}=1}^{N_{c_{1}}}{\overset{`}{\omega }}_{k_{1}}\,{\overset{`}{\omega }}_{k_{2}}\,C\,o\,v\,\left(x_{k_{1}}^{\left(h_{1}\right)},x_{k_{2}}^{\left(h_{2}\right)}\right)={\overset{`}{w}}^{T}\,S\,\overset{`}{w},$$

where *N*_*tr*_ is the number of labeled training trials, the element *S*_*k*_1_,*k*_2__(1≤*k*_1_,*k*_2_≤*N_c_1__*) of *S* is formulated as:

(7) $$S_{k_{1},k_{2}}=\sum_{h_{1},h_{2}=1,h_{1}\neq h_{2}}^{N_{t\,r}}C\,o\,v\,\left(x_{k_{1}}^{\left(h_{1}\right)},x_{k_{2}}^{\left(h_{2}\right)}\right).$$

The optimization problem is defined as:

(8) $$\hat{\overset{`}{w}}=\underset{\overset{`}{w}}{\text{argmax}}\frac{{\overset{`}{w}}^{T}\,S\,\overset{`}{w}}{{\overset{`}{w}}^{T}\,Q\,\overset{`}{w}},$$

under the following constraint:

(9) $$V\,a\,r\,\left(z\,\left(t\right)\right)=\sum_{k_{1},k_{2}=1}^{N_{c_{1}}}{\overset{`}{\omega }}_{k_{1}}\,{\overset{`}{\omega }}_{k_{2}}\,C\,o\,v\,\left(x_{k_{1}}\,\left(t\right),x_{k_{2}}\,\left(t\right)\right)={\overset{`}{w}}^{T}\,Q\,\overset{`}{w}=1,$$

where Var(*z*(*t*)) is the variance of *z*(*t*). Then, for the *n*-th visual stimulus, its optimal task-related filter ${\overset{`}{w}}_{n}$ is the eigenvector corresponding to the largest eigenvalue of $\hat{\overset{`}{w}}$ and its averaged training trials are ${\bar{X}}_{n}$. The *N_f_* task-related filters are applied to their corresponding averaged training trials, i.e., $z={\overset{`}{w}}^{T}\,\bar{X}$, where $\overset{`}{w}=\left[{\overset{`}{w}}_{1},{\overset{`}{w}}_{2},\cdots \,{\overset{`}{w}}_{N_{f}}\right]$ and $\bar{X}=\left[{\bar{X}}_{1},{\bar{X}}_{2},\cdots \,{\bar{X}}_{N_{f}}\right]$. *N_f_* denotes the number of visual stimuli.

Step 2:

Task-unrelated spatial filters $\hat{w}\in {\mathbb{R}}^{N_{c_{2}}\times N_{f}}$ used for a target device can be estimated as $\hat{w}={\left(x\,x^{T}\right)}^{-1}\,x\,z^{T}$, where *N*_*c_2_*_ and *x* ∈ *ℝ*^*N_c_2__*×*N*_*sa*_^ are the number of channels and a single trial in the target device, respectively. Likewise, task-unrelated spatial filters $\hat{w}$ are applied to the single trial *x*, i.e., $\hat{z}={\hat{w}}^{T}\,x$. Obviously, *z* and $\hat{z}$ are the stationary features and the discriminative feature, respectively.

Zheng X. et al. (2020) proposed a cross-task TL approach in MI-based system, which can add more MI tasks based on the traditional ones. For example, the traditional single-limb task, such as left hand (LH) MI, right hand (RH) MI, or foot (F) MI, can be extended to be multiple-limb task, such as both left hand and right hand (LH and RH) MI simultaneously, both left/right hand MI and feet (LH/RH and F) MI simultaneously, etc. To tackle cross-task TL, stationary spatial filters can be first computed for the source domain using CSP. Then, the fishier ratio is used to select discriminative spatial filters for the target domain from stationary spatial filters.

### Parameter Transfer Learning

The goal of PTL is to discover model’s shared parameters from previous source domains (Sannelli et al., 2016).

Zheng M. et al. (2020) proposed a cross-session/subject model using the shared parameters and the specific parameter jointly. First, the source datasets which are most relevant to a new target dataset are selected based on Euclidean distance. Then, the shared parameters $\overset{´}{W}=\left[{\overset{´}{\omega }}_{1},{\overset{´}{\omega }}_{2},\cdots ,{\overset{´}{\omega }}_{s}\right]$ are optimized using *s* selected source datasets as follows:

(10) $$\underset{\overset{´}{W},\overset{´}{\mu },\overset{´}{\Sigma }}{\text{argmin}}\frac{1}{\lambda }\sum_{s}∥X_{s}{\overset{´}{\omega }}_{s}-Y_{s}{∥}^{2}+\sum_{s}\frac{1}{2}[{\left({\overset{´}{\omega }}_{s}-\overset{´}{\mu }\right)}^{T}\Sigma^{-1}\left({\overset{´}{\omega }}_{s}-\overset{´}{\mu }\right)\;+logdet\left(\overset{´}{\Sigma }\right)],$$

where ${\overset{´}{\omega }}_{s}$ denotes the model parameter for the specific source dataset (*X_s_*,*Y_s_*), $\overset{´}{\mu }$ and $\overset{´}{\Sigma }$ are, respectively, the mean and variance of all model parameters, λ refers to a regularization term. After iteratively updating $\overset{´}{\mu }$ and $\overset{´}{\Sigma }$, the shared parameters $\overset{´}{W}$ converges. Finally, the shared parameters and the specific parameter trained in the target dataset are jointly utilized.

Domain adaption is also commonly used in PTL. The model parameter trained in the source domain can be reused and adjusted for the target domain (Wang et al., 2015).

Dai et al. (2019) proposed a domain transfer multiple kernels boosting (DTMKB) framework by applying the boosting techniques for learning multiple kernel-based classifiers. First, these kernel-based classifiers have already trained with the labeled samples from different source subjects. Then, a series of boosting labeled trials from the target subject are input to those classifiers one by one. For each boosting labeled trial, the best kernel classifier is selected and weighted based on the error rate. Finally, all best classifiers and their weights are combined to become an ensemble classifier.

Wei et al. (2018) proposed a cross-subject framework for drowsiness estimation by ranking and fusing different source models. First, for each source subject, an EEG-based drowsiness estimation source model is constructed using PCA and regression. Then, to rank different source models, the following steps are performed:

(1) To estimate the similarity among the source subjects, five distance metrics, including Euclidean distance, correlation distance, Chebyshev distance, cosine distance, and KL divergence, are involved to calculate the distance between subjects’ alert baseline power distributions and construct a multiple distance measurement matrix *M*.
(2) To further evaluate the transferability among source subjects, a transferability matrix *XP* can be obtained by calculating the performance of one source model on another source subject. Both ℳ and *XP* are normalized into z-scores vector by vector.
(3) A linear support vector regression (SVR) model is trained using ℳ and *XP* as the predictor and the response, respectively. For a new target session, the transferability of each source model is estimated based on the trained SVR model using the baseline distances between the corresponding source subject and the target subject. Then, the different source models are ranked from high to low based on the transferability.

Finally, the rankings of all source models are used to generate their weights in a fused model.

Jin et al. (2020) proposed a cross-subject generic model set in P300-based BCI. The P300 signals from 116 source subjects are used to train this generic model set without the participation of the target subject. The initial feature vectors are extracted from these source subjects’ EEG signals. Then, the dimensionality of these feature vectors is reduced using PCA to alleviate computational burden. The resulting feature vectors are grouped into ten clusters using a *K*-means clustering algorithm. Ten weighted LDA (WLDA) classifiers are separately built using the feature vectors from ten clusters. In each WLDA classifier, its discriminant vector is initially trained by traditional LDA classifier using the feature vectors from each cluster. The vectors are then weighted based on their classification accuracies computed in the initial WLDA classifier. Next, the discriminant vector is updated by LDA using the weighted vectors. As a result, a generic model set consists of these ten WLDA classifiers.

Currently, a combination of TL and DL has gained attention in the BCI systems (Fahimi et al., 2019; Bird et al., 2020). TL focuses on the transfer of knowledge between different domains. DL aims to find the inherent knowledge among amounts of labeled trials. If the pre-trained model trained by DL is reliable and adjustable, it is meaningful to transfer the model from one domain to the other one.

Fahimi et al. (2019) developed a cross-subject TL approach based on an end-to-end deep convolutional neural network (CNN) to decode the attentive mental state for EEG-based BCI. The proposed method learns from raw EEG signals to avoid the loss of information and builds a network with the combination of convolutional, max-pooling, and dropout layers using a large scale of EEG data from 120 healthy source subjects. To ensure positive transfer, half of the target subject’s samples are re-trained for adaption by CNN.

The summary of some representative TL approaches is shown in Table 2. In the sixth column of Table 2, ‘Training’ denotes the size of all labeled training sets. The first term of ‘Training’ refers to the size of labeled training set from the target domain, the second term of ‘Training’ refers to the product of the size of labeled training set from one source domain and the number of source domains, whereas ‘Testing’ denotes the size of unlabeled testing set from the target domain. CA denotes the classification accuracy. BCA denotes the balanced classification accuracy. Different from CA, BCA first calculates the classification accuracy for each class and then averages all classification accuracies. CC denotes the correlation coefficient between actual and predicted drowsiness indices.

**TABLE 2:** Summary of representative TL approaches.

Pattern	Type	Domain	TL method	Classifier	Result (training/testing)	References
MI	ITL	Subject	RCSP based on dynamic time warping	SVM	CA: over 75% (10+84 × 8/50)	Azab et al. (2020)
MI	ITL	Subject	A manifold embedded knowledge approach	sLDA	BCA: 68.73% (0+144 × 8/144)	Zhang and Wu (2020)
MI	ITL	Subject	Riemannian procrustes analysis	MDM	CA: 80% (50+200 × 49/150)	Rodrigues et al. (2019)
MI	ITL	Subject	Euclidean alignment TL framework	CSP-LDA	BCA: 73.53% (0+144 × 8/144)	He and Wu (2020b)
MI	ITL	Task	Label alignment TL framework	TS-SVM	CA: over 70% (20+1400 × 1/124)	He and Wu (2020a)
SSVEP	FRT	Device	A task-related component analysis approach	Computing the highest correlation coefficients	CA: over 70% (0+320 × 1/320)	Nakanishi et al. (2019)
MI	FRT	Task	Similar MI tasks transfer based on Fisher ratio	SVM	CA: 81.94% (10+120 × 1/110)	Zheng X. et al. (2020)
Drowsiness	PTL	Subject	A cross-subject framework by ranking and		CC: 0.6448 (20+100 × 8/80)	Wei et al. (2018)
			fusing different source models			
Attentive mental state	PTL	Subject	An end-to-end deep CNN approach		CA: 79.26% (20+40 × 119/20)	Fahimi et al. (2019)

As shown in Table 2, MI-based BCIs have been widely studied in the field of TL. The reason is that there are a lot of publicly available MI datasets. Furthermore, MI EEG signals are evoked by spontaneous movement imagination without external stimulus. Therefore, compared with ERP and SSVEP, MI is more uncontrollable and more eager to minimize the calibration time by using TL methods. In addition, due to effectiveness and convenience, cross-subject ITL approaches are commonly researched. Note that PTL usually fulfills the feature extraction and classification altogether when transferring the model between domains. In terms of results, most TL algorithms can achieve good classification performance (over 70%) using no or few labeled training samples from the target domain.

## Semi-Supervised Learning

### Concepts

Transfer learning can remedy the shortage of the labeled samples from the target domain by importing amounts of labeled data from different source domains. However, it is challenging for TL to cope with huge differences among data available. SSL can make full use of the labeled training samples and unlabeled testing samples from the target subject. Because of fewer data, SSL has less pressure to deal with differences among data than TL.

Semi-supervised learning inherits the merits from supervised learning and unsupervised learning. On the one hand, it is helpful for supervised learning to generate a reliable classifier using amounts of discriminative labeled training samples. Nevertheless, the classification performance of supervised learning decreases sharply with the reduction of labeled training samples. On the other hand, it is of great importance for unsupervised learning to explore the inherent information embedded in amounts of unlabeled testing samples. However, it is difficult to apply unsupervised learning to non-stationary EEG-based BCI. To minimize the calibration procedure, it is crucial for SSL to effectively utilize the labeled training set and unlabeled testing set simultaneously.

In general, SSL methods make additional assumptions on relationship between data distribution and decision function. These include the cluster assumption, the low-density assumption, and the smoothness assumption (Zhu and Goldberg, 2009; Tang et al., 2019). The cluster assumption holds that the samples in the same cluster should belong to the same class. The low-density assumption thinks that the decision boundary should go through the sparse low-density feature space. Both the cluster assumption and the low-density assumption focus on the whole data distribution by adjusting the decision boundary (Fu et al., 2021). However, the smoothness assumption pays more attention to the local data distribution. It is considered that the samples close to each other are likely from the same class (Engelen and Hoos, 2020). Based on such assumptions, different SSL models utilize the training and testing sets in different ways.

(1) Based on the low-density assumption, the transductive SVM (TSVM) model seeks the optimal hyperplane which can go through the low-density area and separate both labeled training and unlabeled testing data with maximum margin (Joachims, 1999).
(2) Based on the cluster assumption, the generative model iteratively performs the expectation step and the maximization step to learn the posterior distributions and optimal parameters for the testing samples surrounding the small training set (Tkachenko and Lauw, 2017).
(3) Based on the cluster assumption, the self-training model usually adopts a specific supervised learning approach as the base classifier to iteratively train the training samples and the extended testing samples with high confidence (Chen et al., 2016; Wang et al., 2017). The extended testing samples come from the testing set. The rest of testing set is usually used to estimate the model’s classification performance. If the size of the extended testing set is 0, the whole testing set is used to build and evaluate the classifier. Likewise, the co-training model iteratively trains two base supervised learning classifiers using each other’s previous classification results (Ren et al., 2014).
(4) Based on the low-density assumption and the smoothness assumption, the graph-based model constructs one or more weighted graphs to analyze the similarity between the training and testing samples and then predicts the classes of the testing samples (Zhao et al., 2015; Gan et al., 2018).

Next, we discuss different SSL models in detail.

### TSVM Model

Transductive SVM was firstly proposed for text classification (Joachims, 1999). Recently, TSVM methods have been gradually used in EEG-based BCI, since SVM is much suitable for EEG signals (Liao et al., 2007; Bi et al., 2019; Xu et al., 2019a).

Liao et al. (2007) introduced a TSVM method for reducing the training effort in EEG-based BCI. A non-linear TSVM based Gaussian kernel approach was proposed to find a more complex structure in the feature space. Particularly, the particle swarm optimization method is used to tune the best parameters for non-linear TSVM.

Bi et al. (2019) developed a prior information-based TSVM (PI-TSVM) method in ERP-based driver-vehicle interfaces. The prior information refers to the ratio of positive samples to negative samples. Its value is set to one to two according to the experimental paradigm. Based on the framework of TSVM, a SVM classifier is trained using the initial labeled training set. The unlabeled testing samples are sorted based on their decision scores in a descending order. Then, using the prior information, the first third and the rest of the sorted testing samples are initially marked as positive labels and negative labels, respectively. To seek optimal hyperplane which can go through the low-density area, a pair of testing samples are repeatedly selected to switch their pseudo labels according to a certain criterion.

Compared with SVM, TSVM approaches have better generalization ability in the case of limited training set. However, they are easily affected by outlier testing samples (Li and Zhou, 2015).

### Generative Model

To our knowledge, there are few literatures about generative model used in EEG-based BCI.

Based on the cluster assumption, Fu et al. (2021) presented a semi-supervised discriminative rectangle mixture approach in MI-based BCI. It is assumed that the prior distribution of decision boundaries is a Gaussian mixture model. Meanwhile, all samples belonging to a specific cluster are supposed within a corresponding decision rectangle. The expectation maximization algorithm is performed to obtain the optimal model by maximizing a posterior estimate for the posterior distributions.

### Self-Training Model and Co-training Model

**FIGURE 4:**
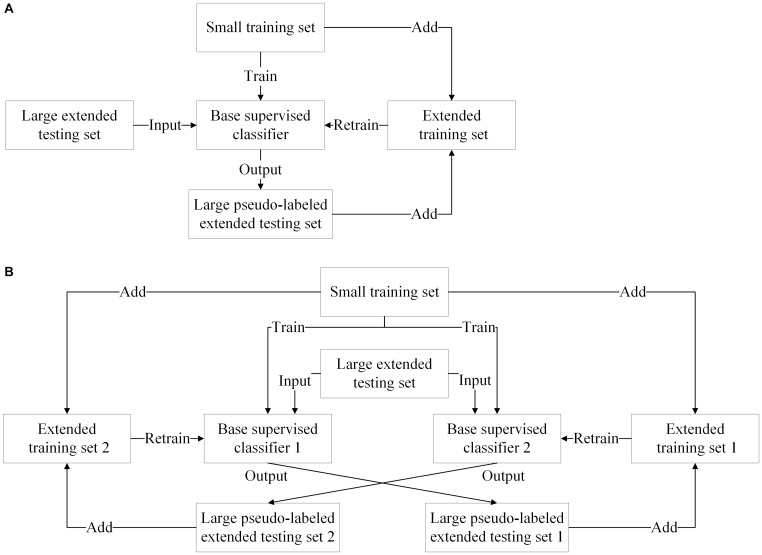
Self-training and co-training models. **(A)** The framework of self-training model. (**B**) The framework of co-training model.

Both the self-training and co-training models are popular because they can take advantage of one or two supervised classifiers. The self-training model iteratively trains a base supervised classifier using the extended training set, whereas the co-training model iteratively trains two base supervised classifiers marking the labels of the extended testing samples for each other. The extended training set is comprised of the extended testing samples from the testing set and the initial training samples. The framework of self-training model and that of co-training model are shown in Figures 4A,B, respectively.

The self-training model can be effectively used for offline and online SSL. In offline SSL, the extended testing samples are obtained *a priori*, whereas in online SSL, they are obtained sequentially and labeled on-the-fly. There are many literatures related to offline SSL, such as TSVM methods, generative methods, and graph-based methods. However, in the online scenario, the self-training methods can expand the training set with newly labeled testing samples and update the classifier along with the adaption of the subject’s mental state.

Based on spectral regression kernel discriminant analysis (spectral regression KDA, SRKDA), Nicolas-Alonso et al. (2015) presented an offline SSL version, namely semi-supervised SRKDA (SS-SRKDA), and an online SSL version, namely sequential updating SS-SRKDA (SUSS-SRKDA). As a base classifier, SRKDA can find optimal projection casting KDA into a regression framework and save the computational cost by Cholesky decomposition. Based on the self-training framework, SS-SRKDA iteratively trains the SRKDA classifier with the training set and the testing set until convergence. SUSS-SRKDA sequentially labels the arriving testing samples using the SS-SRKDA classifier.

The total set can also be divided into the training, validation, and testing sets. To better utilize the unlabeled testing samples, a transductive learning with covariate shift-detection (TLCSD) method was proposed in MI-based BCI (Raza et al., 2016). First, an inductive SVM classifier is trained using the initial labeled training set. The validation set is used to obtain the optimal hyperparameters. Each testing sample is labeled by the trained classifier. Then, for each unlabeled testing sample, TLCSD selects its *K* nearest labeled neighbors and calculates the confidence ratio to judge its usefulness. Finally, both the useful testing sample and its estimated label are added to the existing training set to update the SVM classifier. This process is repeated until all testing samples are processed.

Active learning (AL) is an iterative SSL technique for identifying maximally informative samples. Therefore, it is much suitable for selecting testing samples with high confidence. Marathe et al. (2016) presented an AL implementation in a rapid serial visual presentation (RSVP) paradigm. The proposed approach uses a Query-by-Committee (QBC) framework with a heterogeneous ensemble of popular models, including hierarchical discriminant component analysis (HDCA), CSP+BLDA (CSP-BLDA), xDawn+Bayesian LDA (XD-BLDA). First, the HDCA, CSP-BLDA, and XD-BLDA models are separately trained using the training set. Then, the area-under-curve (AUC) is calculated to measure model efficacy using the validation set. The testing samples are labeled by different models. For each testing sample, its aggregate confidence is calculated based on the distance of each model’s score from the discriminating boundary. The testing samples with the lowest aggregate confidence are chosen to be informative trials and sent to the oracle for labeling. The oracle is assumed to be the user himself.

For co-training model, since the two base classifiers give different separate planes, they are good complements to each other.

Ren et al. (2014) proposed a co-training method based on a biomimetic pattern recognition (BPR) classifier and a sparse presentation (SP) classifier for MI-based BCI. As the base classifiers, both BPR and SR are computationally simple due to few parameters to be tuned. Moreover, they provide reasonably different separate planes, which is important for co-training model. BPR is based on “coverage.” It consists of multiple neurons trained by training data. While SR is based on the compressed sensing theory, which takes advantage of the redundancy in many signals. For different base classifiers, the distance to one class and the residuals of one class are simultaneously considered to select high confident extended testing samples.

Meng et al. (2014) presented a co-training approach combining a LDA classifier with a BLDA classifier. The CSP feature extraction and the co-training classifier are performed jointly and iteratively (Meng et al., 2014). LDA can give good classification performance and fast computational cost simultaneously. While BLDA can give better generalization ability as the Bayesian version of LDA. For different classifiers, the extended testing samples with largest absolute values of classification scores are selected for each other.

Although both the self-training and co-training models are simple and effective, they are susceptible to the incorrect labels of the extended testing samples with high confidence during iterations.

### Graph-Based Model

The graph-based model can construct one or more weighted graphs to reveal the manifold structure behind the training set or the total set. The graph Laplacian is always embedded in the objective function of the SSL classifier.

A semi-supervised quantization approach based on the cartesian *K*-means (SSCK) method was proposed for MI and emotion EEG classification, where the graph Laplacian was built based on the labeled training set and added in the objective function as a manifold regularization term (Liu M. et al., 2020). *L* = *D*−*W* is the Laplacian matrix. Based on the cluster assumption, the entry *W*_*ij*_ of the similarity matrix *W* is assigned a large weight when the *i*-th and *j*-th labeled training samples are in the same cluster, otherwise it is assigned a small weight. *D* is a diagonal matrix with its diagonal elements *D*_*ii*_ = ∑_*j*_*W*_*ij*_. To get more discriminative SSCK model, the following objective function is iteratively optimized:

(11) $$\underset{R,C,B,Y^{\left(l\right)},\mu }{\text{argmin}}(∥R^{T}X_{\mu }^{\left(l\right)}-CY^{\left(l\right)}{∥}_{F}^{2}+\eta ∥R^{T}X_{\mu }^{\left(u\right)}-CB{∥}_{F}^{2}\;+\lambda tr\left(Y^{\left(l\right)}L{\left(Y^{\left(l\right)}\right)}^{T}\right)),$$

where the first and second terms are respectively employed to reduce the squared distortion errors in the transformed training features $X_{\mu }^{\left(l\right)}\equiv X^{\left(l\right)}-\mu \,{\left(1^{\left(l\right)}\right)}^{T}$ and the transformed testing features $X_{\mu }^{\left(u\right)}\equiv X^{\left(u\right)}-\mu \,{\left(1^{\left(u\right)}\right)}^{T}$ by the *K*-means clustering with respect to the rotation matrix *R* and the cluster center *C*. *X*^(*l*)^ and *X*^(*u*)^ are the original training and testing features, respectively. μ is the mean value vector of input data after each iteration. The third term is the supervised Laplacian regularization term. *Y*^(*l*)^ and *B* are separately the training label matrix and the testing label matrix. *tr*(⋅) performs the trace computation. η and λ are the trade-off parameters.

Based on the extreme learning machine (ELM) and deep architecture, a hierarchical semi-supervised ELM (SS-ELM) method was presented for MI task recognition, where the graph Laplacian was calculated using all samples (She et al., 2019a). Firstly, high-level features$G_{\tilde{k}}$, $T_{\tilde{k}}$, and $H_{\tilde{k}}$ are separately extracted from *l* labeled training samples, *u* unlabeled testing samples, and (*l+u)* total samples by deep architecture, where $\tilde{k}$ is the number of hidden layers. Then, these features are input to the SS-ELM classifier to optimize the objective function as follows:

(12) argminβ(∥β∥22+C~⁢∥Gk~⁢β-Y~∥22+λ⁢βT⁢Hk~T⁢L~⁢Hk~⁢β),

where the output weights β can be calculated by Moore-Penrose principle, $\tilde{C}$ is a penalty coefficient on the training errors, $\tilde{Y}$ denotes the labels of training data, λ is a trade-off parameter. $\tilde{L}=\tilde{D}-\tilde{W}$ is the graph Laplacian, $\tilde{D}$ denotes a diagonal matrix, ${\tilde{W}}_{i\,j}$ is the element of the similarity matrix $\tilde{W}$ (1≤*i*,*j*≤*l* + *u*). Finally, ${\tilde{Y}}_{p\,r\,e\,d\,i\,c\,t}=\beta \,T_{\tilde{k}}$ is the labels of testing samples. The proposed approach takes advantage of ELM, deep architecture, and graph-based model.

A *k*-nearest neighbor graph is usually included in the Laplacian matrix to define the relationship among the nearby data points (She et al., 2019b; Peng et al., 2020).

Based on the graph label propagation algorithm and the broad learning system (BLS), a graph-based semi-supervised BLS was proposed in MI-based BCI system (She et al., 2019b). On the one hand, the label information can be smoothed over the graph by the graph label propagation algorithm. On the other hand, BLS has a simpler structure, fewer layers, and a shorter training time than DL. First, the proposed algorithm constructs a *k*-nearest weight matrix $\hat{W}$ as follows:

(13) $${\hat{W}}_{i\,j}=\left\{ \begin{array}{cc} e^{-{∥x_{i}-x_{j}∥}^{2}/\sigma^{2}}\; & \text{if}\,\left\{ \begin{array}{c} x_{i}\in N\,\left(x_{j}\right)\\ x_{j}\in N\,\left(x_{i}\right) \end{array}\right.\\ 0\; & \text{otherwise} \end{array}\right.,$$

where *N*(⋅) is the set of *k* nearest neighbors, σ is a scaling parameter of Gaussian function, *x_i_* and *x_j_* belong to the total set. To obtain the optimal soft label matrix *F*, the objective function is optimized as below:

(14) $$\underset{F}{\text{argmin}}\left(\sum_{i,j}{∥F_{i}-F_{j}∥}^{2}\,{\hat{W}}_{i\,j}+\hat{\mu }\,\sum_{i,j}{∥x_{i}-x_{j}∥}^{2}\,{\hat{W}}_{i\,j}\right),$$

where the first and second terms constrain that the similar trials have similar labels and features, respectively. $\hat{\mu }$ is used to balance the roles of label space *F* and feature space *x*. After optimization, the testing samples are labeled. Then, all features with their labels are successively fed into the feature layer, the enhancement layer, and the output layer of BLS to obtain their prediction labels.

As mentioned above, more than one graphs can be exploited to represent the relationship among all samples.

Combining a label-consistency graph (LCG) and a sample-similarity graph (SSG), a balanced graph-based regularized SS-ELM for MI EEG classification was presented (She et al., 2021). The SSG is the traditional graph Laplacian which reveals the similarity between the training and extended testing data without using the label information (She et al., 2019a). Moreover, to develop the class similarity among the training and extended testing data, the LCG defines a new adjacent graph matrix as follows:

(15) W′i⁢j={1/Nt-t⁢h if⁢both⁢hi⁢and⁢hj⁢belong⁢to⁢the⁢t-th⁢class0 otherwise,

where *h_i_* and *h_j_* are separately the mapping vectors of *x_i_* and *x_j_* represented by the hidden layer of ELM, *N*_*t–th*_ denotes the number of the *t*-th class samples. The pseudo labels of all extended testing samples are obtained by traditional ELM classifier. Then, like the traditional graph Laplacian, the LCG is the new Laplacian matrix *L*′ = *D*′−*W*′, where *D*′ is a diagonal matrix with its diagonal elements D′i⁢i=∑jW′i⁢j. The balanced graph is the linear combination of LCG and SSG with different weights. Finally, such balanced graph is embedded in the objective function of the SS-ELM classifier.

The summary of some representative SSL approaches is shown in Table 3.

**TABLE 3:** Summary of representative SSL approaches.

Pattern	Type	Feature extraction	Classifier	Result [training/validation (extended testing)/testing]	References
ERP	TSVM	Variational autoencoder	A prior information based TSVM method	CA: 85% (30/0/240)	Bi et al. (2019)
MI	Self-training	CSP	Online spectral regression kernel discriminant analysis	CA: 77% (144/0/144)	Nicolas-Alonso et al. (2015)
MI	Self-training	CSP	Transductive learning with covariate shift-detection	CA: 69.72% (240/160/320)	Raza et al. (2016)
RSVP	Self-training	Active learning with an ensemble of models,		AUC: over 0.9 (50%/10%/40%)	Marathe et al. (2016)
		including HDCA, CSP-BLDA, and XD-BLDA			
MI	Co-training	CSP	Both BRP and sparse presentation are assigned as base classifiers	CA: over 70% (14/126/140)	Ren et al. (2014)
MI	Graph	CNN	A semi-supervised quantization approach based on the cartesian *K*-means method	CA: 76.86% (28/0/260)	Liu M. et al. (2020)
MI	Graph	CSP	A graph-based semi-supervised broad learning system	Kappa value: 0.525 (288/0/288)	She et al. (2019b)
MI	Graph	CSP	A balanced graph-based regularized semi-supervised ELM	Kappa value: 0.660 (144/144/288)	She et al. (2021)

As shown in Table 3, most SSL algorithms are applied in the MI-based BCI. Both the self-training and graph-based models are popular. The reason is that the former can take advantage of different base supervised classifiers and then can be easily realized. The latter can directly reveal the sample-similarity or label-similarity among data available. In terms of result, most SSL classifiers can obtain good classification accuracies for two-class problem with the help of a few training samples, the extended testing set or validation set. For multi-class BCI tasks, SSL classifiers can get good kappa values, too.

## A Combination of Transfer Learning and Semi-Supervised Learning

### Concepts

As mentioned above, TL often performs in the feature extraction stage and aims to extract more distinctive target features by transferring the labeled samples from the source domains, whereas SSL often performs in the classification stage and aims to utilize the labeled and unlabeled samples from the target domain. Recently, a combination of TL and SSL has gained an increasing attention (Jiang et al., 2017; Wu, 2017a,b; Zhao et al., 2019; Zhou et al., 2021). To our best knowledge, it occurs in two ways:

(1) A combination of cross-session TL and SSL. For cross-session TL, the samples from the source and target sessions are usually labeled and unlabeled, respectively. They come from the same subject. Therefore, the samples used in cross-session TL are the same to those used in SSL. However, cross-session TL focuses on reducing the discrepancy in data distribution between different sessions, SSL focuses on utilizing the useful information contained in the unlabeled testing data.
(2) A combination of cross-subject TL and SSL. For cross-subject TL, it is a great challenge to deal with high inter-subject variability. For SSL, it is hard to collect amounts of labeled training samples and unlabeled testing samples due to high inter-session variability. After combination, cross-subject TL can relieve the pressure of limited samples for SSL, and SSL can reduce the variability for cross-subject TL since the inter-session variability is generally smaller than inter-subject variability.

Next, we present different kinds of approaches related to the combination of TL and SSL.

### A Combination of Cross-Session TL and SSL

To achieve epileptic seizure classification, Jiang et al. (2017) integrated TL, SSL, and a Takagi-Sugeno-Kang (TSK) fuzzy system. As the base classifier, the TSK fuzzy system can increase the model interpretability for medical diagnostics. Cross-session TL can minimize the differences between the source and target sessions. Based on the cluster assumption, SSL is designed for label clustering. The integrated model aims to seek optimal projection P_*g*_ used in the decision function. The objective function of the proposed model consists of three parts:

Part 1:

To utilize the labeled training samples from the source session, the objective function used for the TSK fuzzy model is defined as follows:

(16) argminPg(12⁢t⁢r⁢((PgT⁢xg,s-ys)⁢(PgT⁢xg,s-ys)T)+λ12⁢t⁢r⁢(PgT⁢Pg)),

where (*x*_*g,s*_,*y_s_*) denotes the new labeled set constructed by Fuzzy C-means (FCM) clustering, λ_1_ denotes a trade-off parameter balancing the complexity of the model and the tolerance of error. The two terms are used to regularize the TSK fuzzy model.

Part 2:

To reduce the differences between the source session (labeled set) and the target session (unlabeled set), the objective function used for cross-session TL is optimized as below:

(17) argminPg(λ2⁢t⁢r⁢(PgT⁢Ω⁢Pg)),

where

(18) Ω=1NT2⁢xg,T⁢[1]NT×NT⁢xg,TT+1NS2⁢xg,s⁢[1]NS×NS⁢xg,sT-1NS⁢NT⁢(xg,T⁢[1]NT×NS⁢xg,sT+xg,s⁢[1]NS×NT⁢xg,TT).

λ_2_ is a regularization parameter. *N_S_* is the size of source session. *N_T_* is the size of target session. *x*_*g,T*_ denotes the new unlabeled testing set from target session constructed by FCM. The term in Equation (17) is used to minimize the projected squared maximum mean discrepancy (MMD) distance between different sessions.

Part 3:

To label the unlabeled testing samples from the target session, the objective function used for SSL is computed as follows:

(19) $$\underset{{\text{p}}_{g}}{\arg\min}\left({\text{λ}}_{3}{\sum_{j=1}^{\hat{C}}\sum_{i=1}^{N_{T}}\hat{\text{μ}}}_{i,j}{\left\|{\text{P}}_{g}{}^{T}{\text{x}}_{gi,T}-{\text{θ}}_{j}\right\|}^{2}\right),$$

where λ_3_ is a regularization term, $\hat{C}$ is the number of clusters, ${\hat{\mu }}_{i,j}$ denotes the label membership of the *i*-th unlabeled sample *x*_*gi,T*_ belonging to the *j*-th cluster, and θ_*j*_ = [0,⋯,0,1_*j*th_,0,⋯,0]^*T*^ denotes a label vector of the *j*-th cluster. The term is used to assign the data close to each other to the same class. Because the optimal P_*g*_ is calculated using the labeled samples from the source session and the unlabeled samples from the target session according to the three parts mentioned above, the objective function is optimized in a semi-supervised way.

The objective function in Equations (16–19) is iteratively optimized to obtain the optimal projection P_*g*_. The proposed model increases the classification performance by integrating the supervised TSK fuzzy system, cross-session TL, and SSL.

### A Combination of Cross-Subject TL and SSL

Compared with the traditional TL methods, the combination of cross-subject TL and SSL adds the unlabeled testing samples from the target subject to build a more subject-specific model.

Originating from a weighted adaptation regularization (wAR) method (Wu et al., 2016), an offline wAR with source domain selection (wARSDS) approach was proposed (Wu, 2017b).

First, the pseudo labels of testing samples from the target subject are initially estimated by the wAR classifier trained using the training samples from the source and target subjects.

Then, the labeled training samples and pseudo-labeled testing samples from the target subject are used to calculate the class centroids of the target subject. To save the computational time, based on *k*-means clustering, most suitable source subjects are selected, whose class distances to the class centroids of the target subject are comparably smaller.

Finally, using all samples from the target subject and selected source subjects, the loss function minimization, the marginal probability distribution adaptation, and the conditional probability distribution adaptation are iteratively performed to update the wARSDS classifier.

Zhao et al. (2019) transferred common spatial filters with SSL for MI BCI. First, for each subject, 30 groups of filters are calculated by CSP after randomly selecting 60 samples per class for 30 times. The representative CSP filters are selected from all filters available based on the clustering method.

Then, all new CSP features from the source and target subjects are generated by projecting these representative CSP filters into the original pre-processed samples.

Finally, the semi-supervised SVM classifier makes full use of the labeled training features from the source subjects and all features from the target subject by optimizing the expectation risk, Tikhonov regularization term, and the manifold regularization. A graph Laplacian with Gaussian kernel function is embedded in the objective function using all features available.

In Table 4, some representative TL+SSL models are briefly listed.

**TABLE 4:** Summary of representative TL+SSL approaches.

Pattern	Type	Feature extraction	Classifier	Result (training/testing)	References
Epileptic	Cross-session TL+SSL	Wavelet packet decomposition/Short time Fourier transform/Kernel PCA	An integrated model combing TL, SSL and the TSK fuzzy system	CA: over 95% (0+150 × 1/50)	Jiang et al. (2017)
MI	Cross-subject TL+SSL	EEGLAB	A weighted adaptation regularization with source domain selection	BCA: about 70% (80+270 × 13/190)	Wu (2017b)
MI	Cross-subject TL+SSL	CSP filters selection based on a clustering method	Semi-supervised SVM	CA: about 70% (40+280 × 4/240)	Zhao et al. (2019)

In the fifth column of Table 4, like Table 2, ‘Training’ refers to the size of all training sets from the target and source domains. Like Table 3, ‘Testing’ refers to the size of testing set from the target domain. As shown in Table 4, a combination of TL and SSL is effective since their classification performance can also reach 70% on average using few training samples from the target domain. Therefore, the combination of TL and SSL will be promising in future.

## Discussion

Based on many papers surveyed herein, we briefly summarize various signal processing approaches to reduce calibration effort in EEG-based BCI.

(1) In terms of EEG-based BCI
  Compared with the traditional EEGs, such as SSVEP and P300, MI EEG is more widely studied in the small training set scenario. The reason is that there are numerous MI datasets available. Moreover, MI is harder to control for both healthy and unhealthy subjects. It is more urgent for MI to minimize calibration time. Thus, many base feature learning and classifier learning methods applied to MI are always referred, such as CSP, LDA, SVM, MDM and so on. Additionally, drive drowsiness and emotion state estimations also need to shorten calibration effort for their complex BCI tasks. Similar methods can be used to solve this problem, such as ensemble models, CNN, etc.
(2) In terms of TL
  Compared with SSL, TL is more popular regardless of high inter-domain variability. The reason is that TL provides more sufficient samples from the source domains than SSL and promotes the development of generic model.
  As shown in Table 2, Most TL approaches can reach satisfied classification performance (over 70%) even if the calibration time is reduced or suppressed. For a calibration-free TL model, unlabeled samples from the target domain can also build a subject-specific model.
  Currently, most TL approaches aim to adapt the source domains to the small target domain. However, if the distribution of the small target domain is not discriminable, the classification performance might decrease sharply after such adaptation. To our best knowledge, few literatures cope with this problem. In our opinion, flexible measures should be taken according to the discriminability of the small target domain. Moreover, most TL approaches are not suitable for online BCI since they are designed offline.
  Data alignment-based instance transfer is very promising since it can make aligned covariance matrices from different domains comparable. However, high dimensionality of covariance matrix may lead to curse of dimensionality. Therefore, channel selection is a basic problem for data alignment-based TL. Unsupervised PCA and supervised CSP can be used to cope with this problem.
  Deep learning is highly effective using the big training set. However, it cannot be directly used in the small training set scenario. Thus, in the PTL, DL can build a pre-trained model using amounts of samples from source subjects and then adapt the model for the new target subject. CNN is widely used in EEG-based BCI.
(3) In terms of SSL
  Although few SSL literatures are proposed to shorten the calibration procedure. SSL classifiers still outstand themselves by discovering the useful information embedded in the labeled and unlabeled samples. In EEG-based BCI, the self-training model can be easily achieved by using the well-known supervised model as the base classifier. Additionally, the graph-based model is popular by providing the overall view based on the total samples.
  As shown in Table 3, compared with TL approaches, most SSL approaches use more training samples from the target domain to obtain good classification performance. Therefore, it is further testified that the number of samples available is extremely important to reduce calibration time and achieve good performance.
  Most SSL classifiers surveyed in this paper are designed offline. However, the EEG-based BCI is fundamentally online. Self-training SSL model is suitable for the online scenario. It should be combined with other SSL models to adjust their classifiers in real time.
(4) In terms of the combination of TL and SSL
  The combination of TL and SSL is emerging and promising since it not only relieves the pressure of limited sample, but also brings more subject-specific samples. It should be further explored to increase its effectiveness.
  As shown in Table 3, although few approaches relate to such combination, they still achieve good performance (over 70%) with few samples from target domain by taking advantage of TL and SSL.
(5) In terms of all signal processing approaches
  To reduce the calibration time, we focus on the signal processing approaches in this paper. Nevertheless, the BCI performance also relies on the subject, the BCI paradigm, and so on. So far, fewer literatures discuss them altogether. In our opinion, different subjects have extremely different performance in EEG-based BCI. Therefore, different signal processing approaches should be designed for different subjects. In addition, more friendly BCI paradigm should be used for better experimental experience.

## Conclusion

In this study, we have surveyed the signal processing approaches to minimize the calibration procedure in EEG-based BCI. The numerous approaches in this paper can be divided into three main categories: TL, SSL, and a combination of TL and SSL.

We can draw the following conclusions, which may inspire future research directions:

(1) It is more difficult to decode MI EEG than SSVEP, P300, etc. MI EEG-based BCI should be further studied to speed up its application.
(2) TL is instrumental to reduce the calibration time. Data alignment-based TL is a good choice to relieve the differences between domains. TL can be combined with SSL or DL to achieve a calibration-free model.
(3) SSL can build a more subject-specific model than TL. Currently, offline SSL classifiers have been paid more attention than online SSL classifiers. In terms of online BCI control, online SSL classifiers should be further researched.
(4) The performance of EEG-based BCI is closely related to the signal processing approaches, the BCI subject, the paradigm, and so on. Most signal processing approaches in this paper are evaluated offline based on the publicly available BCI datasets. Future signal processing approaches should further adapt to the mental state change of the BCI subject in real time and work using a more friendly paradigm.

## Author Contributions

YX designed the study. XH and YX wrote the manuscript. JH and WY collected the relevant literatures. HY and RH prepared the figures. SW reviewed and edited the manuscript. All authors read and approved the submitted manuscript.

## Conflict of Interest

The authors declare that the research was conducted in the absence of any commercial or financial relationships that could be construed as a potential conflict of interest.

## Publisher’s Note

All claims expressed in this article are solely those of the authors and do not necessarily represent those of their affiliated organizations, or those of the publisher, the editors and the reviewers. Any product that may be evaluated in this article, or claim that may be made by its manufacturer, is not guaranteed or endorsed by the publisher.
